# Risk Factors for Childhood Leukemia: Radiation and Beyond

**DOI:** 10.3389/fpubh.2021.805757

**Published:** 2021-12-24

**Authors:** Janine-Alison Schmidt, Sabine Hornhardt, Friederike Erdmann, Isidro Sánchez-García, Ute Fischer, Joachim Schüz, Gunde Ziegelberger

**Affiliations:** ^1^Department of Effects and Risks of Ionizing and Non-ionizing Radiation, Federal Office for Radiation Protection (BfS), Neuherberg, Germany; ^2^Division of Childhood Cancer Epidemiology, Institute of Medical Biostatistics, Epidemiology and Informatics (IMBEI), University Medical Center of the Johannes Gutenberg University Mainz, Mainz, Germany; ^3^Environment and Lifestyle Epidemiology Branch, International Agency for Research on Cancer, World Health Organization (IARC/WHO), Lyon, France; ^4^Experimental Therapeutics and Translational Oncology Program, Instituto de Biología Molecular y Celular del Cáncer, CSIC/Universidad de Salamanca, Salamanca, Spain; ^5^Department of Pediatric Oncology, Hematology and Clinical Immunology, Medical Faculty, Heinrich Heine University, Düsseldorf, Germany

**Keywords:** magnetic fields, genetic susceptibility, environmental exposure, acute lymphoblastic leukemia, childhood leukemia, risk factors, radiation

## Abstract

Childhood leukemia (CL) is undoubtedly caused by a multifactorial process with genetic as well as environmental factors playing a role. But in spite of several efforts in a variety of scientific fields, the causes of the disease and the interplay of possible risk factors are still poorly understood. To push forward the research on the causes of CL, the German Federal Office for Radiation Protection has been organizing recurring international workshops since 2008 every two to three years. In November 2019 the 6th International Workshop on the Causes of CL was held in Freising and brought together experts from diverse disciplines. The workshop was divided into two main parts focusing on genetic and environmental risk factors, respectively. Two additional special sessions addressed the influence of natural background radiation on the risk of CL and the progress in the development of mouse models used for experimental studies on acute lymphoblastic leukemia, the most common form of leukemia worldwide. The workshop presentations highlighted the role of infections as environmental risk factor for CL, specifically for acute lymphoblastic leukemia. Major support comes from two mouse models, the *Pax*5^+/−^ and Sca1-*ETV6-RUNX1* mouse model, one of the major achievements made in the last years. Mice of both predisposed models only develop leukemia when exposed to common infections. These results emphasize the impact of gene-environment-interactions on the development of CL and warrant further investigation of such interactions — especially because genetic predisposition is detected with increasing frequency in CL. This article summarizes the workshop presentations and discusses the results in the context of the international literature.

## Introduction

Leukemia is the most frequent cancer in children, with a proportion of about 30% of all cancers diagnosed in children before the age of 15 years (1). The most common form, lymphoid leukemia, makes up one fourth of all cancers. Almost 98% of childhood lymphoid leukemias are precursor cell leukemias with precursor B-cell acute lymphoblastic leukemia (pB-ALL) being the most common form (1). Over the past decades, advances in diagnostics, risk grouping, pharmacology, and treatment combinations have led to remarkable enhancements in treatment of and survival from childhood leukemia (CL), with an overall survival for acute lymphoblastic leukemia (ALL) exceeding 90% in high-income countries nowadays (2). However, knowledge of the complex causes of the disease that may help implementing preventative measures, is still lacking. A growing body of research has targeted a wide range of potential risk factors for childhood ALL, including genetic and environmental ones (3). Environmental risk factors, as defined in this review, include exposure to environmental pollutants, such as air pollution, life style factors, such as parental tobacco smoking or alcohol consumption, microorganisms and viruses, and natural as well as man-made exposures like radiation. Two observations in particular attracted the attention of the German Federal Office for Radiation Protection (Bundesamt für Strahlenschutz, BfS): a moderately increased risk of CL (including ALL) from exposure to extremely low-frequency magnetic fields (ELF-MF) (4), like from power lines, and an increased incidence of CL (including ALL) near German nuclear power plants (NPPs) (5). Both findings are difficult to explain given the current knowledge of the biological mechanisms. Non-ionizing radiation deposits too little energy in cellular DNA and other likely targets to be considered directly causative. While ionizing radiation is an established cause of CL, exposures in the vicinity of NPPs are too low to recognize a direct causal relationship. Motivated by these observations the BfS set up a research agenda and corresponding research recommendations on the basis of two international workshops held in 2008 and 2010 that brought together an interdisciplinary group of experts (6). The research recommendations were last updated on a third workshop in 2012 (7), and two follow-up meetings were organized subsequently in 2016 and 2019. In some research areas the increasing research efforts made some particular progress and new results appear to answer at least some of the open questions that were addressed in the research recommendations set up in 2012. This article summarizes the latest evidence and new findings that were presented at the 2019 International Workshop on the Causes of CL held in Freising, embedded in a discussion in the context of the international literature.

## Incidence and Time Trends

In 2018, the International Agency for Research on Cancer (IARC) in collaboration with the International Association of Cancer Registries (IACR) coordinated a huge effort to assess the most recent incidence of childhood cancer worldwide based on quality-assured data collected from cancer registries: International Incidence of Childhood Cancer volume 3 (IICC-3), which is at the present time the most comprehensive and most up-to date source of data on global childhood cancer incidence (8, 9). Results of IICC-3 and a comparison of the data with incidence rates from 1980s were presented at the workshop. The IICC-3 study included data from 2001–2010 on cancer in children and adolescents diagnosed before 20 years of age in populations covered by cancer registries that met predefined data quality criteria. Leukemia was the most common cancer worldwide representing 36.1% of all cases in children aged 0–14 years and 15.4% in adolescents aged 15–19 years (8). The latest internationally comparable data on incidence patterns of childhood cancer have been published in IICC-2 in 1998 (10) covering (approximately) the decade of the 1980's. Regarding all childhood cancers combined, the assessment of time trends in the incidence rates between the two time periods, 1980's and 2001–2010, revealed an overall increase in registered childhood neoplasms, from 124.0 to 140.6 per million person-years since the 1980's. The increase was seen worldwide except for the sub-Saharan Africa region, where in fact a decrease in registered childhood cancers was observed. Looking specifically on time trends for lymphoid leukemia, a similar picture was evident with increasing incidence rates across regions. The increase was particularly pronounced in sub-Saharan Africa (in contrast to the decrease in all childhood cancers combined) and North Africa where the age-standardized incidence rates had doubled. An increase in lymphoid leukemias was also seen in South, Southeast, and West Asia as well as in Eastern Europe, albeit less pronounced (11). Improvement in diagnosis and registration might likely explain some of the increase of incidence rates since the 1980's. Notably, the IICC-2 and IICC-3 studies were restricted to cases diagnosed and treated within the national health care system, naturally missing undetected cases and those without a verified diagnosis (12). Major under-ascertainment in cancer registries was identified for childhood cancer, particularly in low- but also middle-income countries, because a significant fraction of suspected cases never reaches complete diagnosis and treatment. The latter is mainly due to related costs or lack of infrastructure (12). This is why two independent studies projecting the more realistic true childhood cancer incidence came up with numbers 70–80% higher than those collected by the cancer registries (13, 14). CL appeared to be among the childhood cancer types even more underestimated than many solid cancers with more visible symptoms (15).

At this stage, it is impossible to disentangle the different factors contributing to geographical differences and differences in time trends. Completeness of ascertainment is certainly a major factor. However, it might also reflect differences in susceptibility to leukemia in different racial or ethnic groups as seen in the IICC-3 data, where the highest leukemia rates in the USA were seen in White Hispanic children whereas it was less common in US Black children (8). Exposure to environmental factors may also differ between low-, middle- and high-income countries. To solve these issues global research efforts are essential.

## Genetic Risk Factors

### Preleukemic Clones

pB-ALL shows several biologically distinct subtypes defined by chromosomal alterations of which the most frequent are aneuploidy and chromosomal translocations (16). Strong evidence for a prenatal origin of aneuploidy and of several chromosomal translocations came from studies of concordant leukemia in monozygotic twins and from screening of Guthrie cards (17–19). Subsequent studies examined if the most frequent translocation that leads to the formation of the *ETV6-RUNX1* fusion gene can also be found in healthy newborns. These studies were summarized and discussed at the workshop. Using cord blood samples from healthy newborns the presence of *ETV6-RUNX1* gene fusions was previously revealed in around 1–2% of samples employing RNA as a specimen and RNA-based detection methods such as RT-PCR (20–23). Using a novel detection method called GIPFEL (“Genomic inverse PCR for exploration of ligated breakpoints”) that utilizes DNA as starting material (24), Schäfer et al. (25) found a frequency of 5% for the *ETV6-RUNX1* fusion gene in cord blood of healthy newborns in a Danish cohort. The use of a more reliable DNA-based approach, instead of the previously used RNA-based approaches, is a likely cause for the different results regarding *ETV6-RUNX1* frequency in newborns (26). Recently, the *TCF3-PBX1* fusion, present in around 5–10% of B-ALL patients and assumed to arise postnatally, has been detected in 0.6% of umbilical cord blood samples of healthy newborns by GIPFEL, confirming that this translocation can likewise occur prenatally (27).

The frequency of fusion genes in healthy newborns exceeds by large (≥100 times) the incidence of the corresponding leukemia subtypes and might still be underestimated because the detection rate of GIPFEL is <100% (24, 27). An alternative explanation for this high frequency of fusion genes in healthy newborns might be that the *ETV6-RUNX1* fusion gene detected in healthy individuals is only present in non-self-renewing differentiated cells, and not in hematopoietic stem cells or early progenitors, which are known to be more capable of developing malignant clones (28). Therefore, second hits are clearly needed for progression of these preleukemic clones into full-blown leukemia. However, it is currently not known how much the frequency of the *ETV6-RUNX1* fusion genes differs across infant populations around the world, and future studies have to show how the presence and frequency of preleukemic clones in blood at birth affects the risk of the newborn for developing ALL later in life. Revealing the changes caused by chromosomal translocations, such as *ETV6-RUNX1*, and leading to progression of the disease, could then potentially allow to prevent pB-ALL development in preleukemic carriers (29).

### Inherited Genetic Susceptibility

The current state of knowledge on inherited genetic susceptibilities was summarized in a talk making clear that germline predisposition is more and more recognized as an important risk factor for the development of childhood ALL (30). Genome-wide association studies have identified susceptibility loci in *ARID5B, CEBPE, BMI1, CDKN2A/2B* and others that are associated with an increased risk to develop childhood ALL [for a summary see (31)]. Some of these are more frequently associated with certain racial and/or ethnic groups; e.g., *ARID5B* is more frequently found in Hispanics (32) and might be a possible explanation for the higher incidence rates seen in this ethnic group (see section Incidence and Time Trends). Rare germline mutations in developmental hematopoietic genes, like *ETV6, PAX5* or *IKZF1*, have also been shown to predispose children to ALL (30). Rare syndromes, like Cornelia de Lange and Rubinstein-Taybi syndrome that have been associated with childhood ALL, further point to a connection between variants in cohesin complex genes and CREBBP/EP300 pathway, respectively, and ALL susceptibility (33, 34). Other syndromes connected to a higher risk of ALL and AML include Down syndrome (35), Noonan syndrome (36), constitutional mismatch repair deficiency syndrome (37), Fanconi anemia, and others summarized in (30, 38). The total prevalence of pathogenic germline mutations in known cancer predisposing genes in children and adolescents with leukemia is 4.4%, but this is probably only the tip of the iceberg (39). Novel techniques used for testing for hereditary cancer predisposition syndromes (CPSs), in particular whole-exome sequencing of parent-child trios, lead to the discovery of new germline risk variants (40–42). Trio sequencing can also identify new inheritance patterns in children with cancer where the family history is unremarkable and does not point to an underlying CPS. This is the case with a so called digenic inheritance pattern, when two germline variants in two different genes are needed for causing the clinical cancer phenotype. One mutation is inherited by each of the unaffected parent, or, alternatively, one occurs *de novo* (43). Thus, such di- or oligogenic inheritance patterns could be accountable for a substantial number of childhood cancers (44). The use of trio sequencing can therefore give important insights into the mutational landscape of CPSs as well as into the mechanisms of cancer development in children (43). Expanding this knowledge is a crucial step toward targeted treatments as well as precision-prevention programs (42). Nevertheless, testing for CPSs can provoke emotional and relational challenges in the families in addition to the distress of the child's cancer diagnosis and treatment (45).

### Epigenetics

Global epigenetic changes are a hallmark of cancer, and genetic as well as metabolic and environmental stimuli can cause such changes (46, 47). As epigenetic modifications are also characteristic for childhood ALL (48), one session focused specifically on this topic. A summary of the evidence of epigenetic priming in cancer cells was presented. Epigenetic priming is based on the suggestion made by several studies that oncogenic lesions in human cancers contribute to cancer development by (epi-)genetically modifying the cancer-initiating cell but are dispensable for tumor progression (49, 50). In hematologic malignancies such epigenetic reprogramming has been linked to hematopoietic stem/progenitor cells (51, 52). The epigenetic modifications are preserved throughout tumor development, even if the oncogene is no longer present or expressed, and may remain latent until triggered by either endogenous or environmental stimuli (49). These stimuli, or second hits, can arise randomly. However, the epigenetically primed cell may also be more sensitive to a certain environmental exposure that will favor the emergence of a second hit. In other cases, exposure to an environmental factor could cause epigenetic changes that increase the susceptibility for secondary genetic alterations. This is described as a gene-environment-interaction (49). A good example is pB-ALL. Here it was assumed that, e.g., the frequent chimeric transcription factor *ETV6-RUNX1* epigenetically primes an uncommitted cell subset thereby inducing an aberrant B cell differentiation program that is later on susceptible to transformation (53). Evidence for this came from a transgenic *ETV6-RUNX1* mouse model, described in more detail in section ‘Epidemiological Evidence for an Infection-Mediated Childhood Leukemogenesis', where predisposed mice only developed leukemia after exposure to infections (54).

Inducing DNA methylation changes may also be a mechanism of action for at least some of the environmental risk factors that have been associated with CL (see section Environmental Risk Factors). For example, given the current knowledge of the mechanism of action of ELF-MF, this type of exposure cannot damage the DNA directly but might induce epigenetic changes that could affect the child's risk for CL later in life (55). Several large-scale epigenome-wide association studies have reported associations of relevant maternal exposures during pregnancy, including tobacco smoking, air pollution, and body mass index, with DNA methylation in offspring neonatal blood. For instance, decreased methylation at aryl-hydrocarbon receptor repressor (*AHRR*) CpG cg05575921 has been associated with exposure to maternal smoking during pregnancy (57). Such methylation changes could affect health later in life (56). If this would be the case for ALL, common DNA methylation changes should be associated with both a given exposure and leukemia. This hypothesis, formulated and investigated by Timms et al. (58) using a “meet in the middle” approach, was discussed at the workshop. Briefly, genome wide DNA methylation changes after exposure to environmental risk factors associated with CL were compared to ALL-specific methylation changes. Overlapping gene loci were found for several risk-associated exposures, including maternal radiation exposure, alcohol intake, sugary caffeinated drink intake during pregnancy, and smoking. For radiation exposure, alcohol intake, sugary caffeinated drinks, and attended day nursery, more than 70% of the gene loci that overlapped with ALL-specific gene loci also had the same direction of methylation change, i.e. were hypo- or hyper-methylated (58).

Investigating DNA methylation changes in response to environmental risk factors seems to be an appealing method for studying the contribution and mechanism of action of these factors in promoting disease development (59). Using epigenetic biomarkers for maternal smoking during pregnancy, i.e., DNA methylation at *AHRR* and a recently established polyepigenetic smoking score, two recent studies provided evidence that prenatal tobacco smoke exposure was associated with a higher frequency of somatic gene deletions among childhood B-ALL cases (60, 61). However, these results are inconsistent to epidemiological findings because those studies did not show an association between maternal smoking and childhood ALL risk (62). Epidemiological evidence for other parental exposures, like maternal caffeine intake or alcohol intake, is likewise inconsistent (55). More studies are therefore needed to understand the induction of epigenetic changes by environmental exposures and especially their contribution to CL development.

## Environmental Risk Factors

### Infections

In the context of infections and leukemia there are two hypotheses that have to be mentioned shortly. The first one is the “population mixing” hypothesis by Kinlen (63). The second one is Greaves “delayed infection” hypothesis (64). Both hypotheses propose that ALL is a consequence of an abnormal response to common infections. Currently, three additional models of ALL evolution exist, which also point to infection-induced immune disturbances as being responsible for leukemia evolution as recently summarized by Hauer et al. (65). A more detailed view into the role of infections is given elsewhere (17, 66, 67).

#### Epidemiological Evidence for an Infection-Mediated Childhood Leukemogenesis

Indications for an infectious etiology of CL came from observations of leukemia cases occurring in closer spatial and temporal proximity than would be expected if they occurred independent from one another. The first systematic review and pooled analysis of such space-time clustering studies was presented and discussed at the workshop. This study showed strong evidence of clustering of CL at time of diagnosis for children aged 0–5 years, an age range including the peak incidence for leukemia at 2–4 years (68). Results were similar for ALL. Such clustering in space and time could be explained by “mini-epidemics” of a single infection leading to local clusters of leukemia cases (68), which are observed from time to time (69–72). For children aged 5 to 15 years no clustering at both birth and diagnosis was observed, and results for lymphoma and CNS tumors provided only weak evidence for space-time clustering (68). Nevertheless, the systematic review was restricted to studies using a certain methodology only and there have been other approaches showing no evidence for clustering, e.g., in Germany (73, 74). Studies on population mixing showed evidence for an excess risk of CL in studies of extreme population mixing rather than in studies with modest one (75). On balance, there is some evidence for clustering but it is not fully understood to what extent and under which circumstances it occurs.

A critical time window for infections and risk of leukemia is not only postnatally in early childhood but includes also the time during pregnancy. Maternal infections during pregnancy have long been studied as potential risk factors for CL and the epidemiological evidence was lastly summarized by Maia Rda and Wünsch Filho in 2013 in a narrative review (76). Only recently, He et al. (77) published the first systematic review and meta-analysis on this topic including 15 studies on ALL and 14 on CL and the results were discussed at the workshop. The most frequently studied infection variables for ALL as well as CL were viral or virus-associated infections, followed by systemic symptoms (e.g., urinary tract infection), bacterial infections, and fungal infections. Most of the studies reported a positive association of CL and ALL with infection. When looking for specific types of infection a higher risk for ALL was associated with influenza infections, whereas CL in general was associated with influenza, rubella, and varicella infections during pregnancy. Nevertheless, the authors report high heterogeneity across the studies and an insufficient number of studies, which is why the results should be handled with caution. A major limitation relates to the self-reported information assessed years after the pregnancy that most studies rely on (77). The same group very recently published a pooled analysis of six population-based birth cohorts using prospective data (78). Birth cohorts are less prone to recall and selection bias than case-control studies that were predominantly included in the previous meta-analysis. Results of the pooled analysis showed a higher risk for any leukemia (including ALL) in association with maternal urinary tract infections as well as respiratory tract infections during pregnancy; in contrast to the meta-analysis that did not find such an association. The association with influenza infections was again observed but the effect size was lower compared to the meta-analysis (78).

#### Experimental Evidence for an Infection-Mediated Childhood Leukemogenesis

Experimental evidence that infections promote pB-ALL development comes from a new pB-ALL mouse model that was presented in a special session on mouse models (see also section Experimental Findings): Transgenic Sca1-*ETV6-RUNX1* mice only developed pB-ALL when exposed to common pathogens (conventional facility, CF conditions) but not when kept in a specific pathogen-free (SPF) environment (54). In the Sca1-*ETV6-RUNX1* mouse model, human *ETV6-RUNX1* is expressed within hematopoietic stem/progenitor cells thereby mimicking human *ETV6-RUNX1* preleukemic biology. After exposure to an infectious environment these mice show a significant increase in pro/pre-B cells, although differentiation to mature peripheral blood B-cells is not impaired. Furthermore, when housed under CF conditions, these mice showed a distinct expression pattern, compared with healthy age-matched wild type mice, with significantly higher expression of recombination activating gene 1 (*Rag1*) and *Rag2* and differential regulation of epigenetic regulator genes of the lysine demethlyase (KDM) family (54). Similar observations were made with the *Pax5* heterozygous knock out mouse model where mice only developed pB-ALL under CF conditions (79). Susceptibility to infections in *Pax*5^+/−^ mice was suggested to be due to a higher sensitivity of *Pax*5^+/−^ pro-B cells to interleukin 7 (IL-7) withdrawal, favoring the accumulation of secondary *Jak3* mutations as a rescue mechanism in these mice (54, 79).

It has been suggested that infections trigger pB-ALL development by induction of the mutagenic enzyme activation-induced cytidine deaminase (AID), which is normally involved in producing antibody diversity. AID may promote secondary genetic changes in preleukemic B-cell precursor cells but evidence for this view mainly came from *ex vivo* studies (80, 81). To overcome this restriction, the role of AID was examined in crossed *Pax*5^+/−^ mice by a gain- and loss-of-function experiment, respectively (80). The presented results of this study clearly showed that genetic deletion of AID does not affect the latency and penetrance of pB-ALL and premature expression of AID in earliest pro-B-cell stages does not promote pB-ALL development. Additionally, AID expression was not observed in preleukemic precursor B-cells of Sca1-*ETV6-RUNX1* or *Pax*5^+/−^ mice held in SPF or CF conditions, respectively. These results confirm that infectious stimuli can promote malignant B-cell leukemogenesis through AID-independent mechanisms (80).

Another talk provided new hints that might explain how genetic predispositions affect the susceptibility to infections and in turn promote leukemia. This involves namely the gut microbiome which was shown to differ between *Pax*5^+/−^ mice and WT mice under SPF and CF housing conditions (82). It is well known that microbes colonizing the gastrointestinal tract are integral in shaping the development and function of the immune system and alterations in the composition of the microbiota have been linked with several human diseases (83). To determine its role in pB-ALL development, the gut microbiome of *Pax*5^+/−^ mice was depleted with antibiotics by a short-term treatment (for 8 weeks) starting when mice reached adulthood. When kept constantly under SPF conditions, i.e. without an infectious stimulus, 50% of treated *Pax*5^+/−^ mice developed pB-ALL whereas untreated mice did not. It was further shown that the composition of the gut microbiome varied between Pax5^+/−^ mice, which stayed healthy, and *Pax*5^+/−^ mice, which developed leukemia, but a specific microbe connected with the development of pB-ALL could not be identified. The observation that predisposed mice with a depleted gut microbiota developed leukemia, even without infectious stimuli, suggests that an intact gut microbiome protects genetically predisposed mice from developing pB-ALL (82). This protective effect is likely mediated through the release of microbial components or metabolites, or direct microbial binding to Toll-like receptors on innate immune cells (65, 67).

Further insights into how infections could promote leukemia development came from a presented co-culture study of murine *ETV6-RUNX1*-positive Ba/F3 pro-B cells and bone-marrow mesenchymal stromal cells (BM-MSCs). In a competitive growth assay, *ETV6-RUNX1*-positive and control Ba/F3 cells were mixed and plated on murine BM-MSCs with or without IL-6/IL-1β/tumor necrosis factor-alpha pro-inflammatory cytokines, which are normally secreted by pathogen receptors expressing cells in response to several types of infections (84). This mesenchymal inflammatory environment or “niche” favored the emergence of *ETV6-RUNX1*-positive Ba/F3 clones by differentially affecting their proliferation and survival. Moreover, this inflammatory niche preferentially attracted *ETV6-RUNX1*-positive Ba/F3 clones through the CXCR2 receptor and increased the extent of DNA double strand breaks as judged by the levels of histone AX phosphorylation (γH2AX), thereby providing a chance for transformation of the pre-leukemic clone (84).

Taken together, the role of infections as risk factor in the etiology of ALL, specifically pB-ALL, has strengthened considerably since the last research recommendations in 2014 (7), with the strongest support coming from the *Pax*5^+/−^ and Sca1-*ETV6-RUNX1* mouse models. Therefore, training the immune system early in life could potentially help to prevent leukemia development and several epidemiological studies demonstrate that proxies for infectious agents or immune challenges early in life reduce the risk of ALL (65). Questions remain to be clarified regarding the time window in which infections are relevant, the specific infectious pathogen or pathogens that are involved (if any single given microbe could in fact be responsible) as well as the mechanism(s) and pathway(s) by which infections drive – or protect against – ALL development. The ongoing coronavirus disease 2019 (COVID-19) pandemic is affording an opportunity to answer some of the open questions, and is putting Greaves delayed infection hypothesis to the proof: Children all over the world did not encounter SARS-Cov-2 (severe acute respiratory syndrome coronavirus type 2) before, and the lockdown measures might have led to far fewer encounters with pathogens in infancy than usual (85). In the next years, it should be thoroughly investigated if this will lead to a higher risk of ALL in children who experienced lockdown measures.

### Ionizing Radiation

High and moderate doses of IR have been well recognized as environmental risk factor for CL for several decades. Exposure during young ages seems to be particularly critical, as study findings are consistent with a higher risk of radiation-induced cancer after exposure during childhood, compared to exposure later in life (86–90). The dose response for leukemia after IR exposure is described as linear-quadratic, slowly changing at low doses, but rapidly at high doses (91). So far, generally, the linear no-threshold model for radiation effects is widely accepted by national and international bodies for assessing the risks resulting from exposures to IR (92), but this means that effects in the low dose range - defined as doses <100 mGy absorbed dose (93) - are often extrapolated. This is also true for leukemia models. This low dose range is most relevant for the general population. However, low-dose studies are a challenge due to the need of large cohorts and high individual variation. Therefore, evidence for leukemia in the range of low doses is still sparse. Strongest support for the risk of CL comes from pooled analyses from exposure for diagnostic or therapeutic reason. Cumulative active bone marrow (ABM) doses between 100 and 20 mSv (effective dose) in childhood/adolescence increased the risk significantly (94). A recent study of six pooled studies assessing cancer risks associated with computed tomography (CT) with available ABM between 5.9 and 10.1 mGy found a significant increased risk for CL (95). To date, no increased risk was found for single X-ray examinations (95, 96).

Sources of low doses of IR not only include exposure by medical radiation but also man-made environmental exposure (e.g., nuclear weapons testing). Attention was drawn on several statistical associations of CL in the vicinity of nuclear installations. On a previous workshop in 2012, the consistent findings and trends in European studies about CL risk near NPPs were discussed in the context of previous findings, leukemia etiology, and other risk factors (7). It was concluded that there was no elevated risk of CL globally near NPPs in children <15 years old. However, there might be some elevated risk of CL when considering the 0–4-year age category within 5 km from a NPP, even though the associations were not statistically significant. Recent data from Belgium could link the leukemia risk of young children only to one specific site (97).

By far the greatest contribution to exposure received by the general world population comes from natural background radiation (NBR) (98). Numerous epidemiological studies investigated the association of NBR exposure (including radon and gamma radiation exposure) and cancer risk (including CL), but results are mostly inconsistent (99). In a special workshop session dedicated to NBR, a recent study from Switzerland was discussed that used data from a census-based cohort study to check for an association between cancer in children <16 years of age and exposure to terrestrial gamma and cosmic radiation. The study found evidence of associations for leukemia and CNS tumors with a hazard ratio of about 1.04 per mSv cumulative whole-body dose for both groups (100). However, biases due to inaccurate exposure assessment could not be excluded and statistical power was limited due to small sample sizes. A very recent study considered more accurate measurements of terrestrial radiation based on a new map of terrestrial radiation in Switzerland and an extended cohort. The authors confirmed the recent results that NBR contributes to the risk of leukemia in children (101). However, studies in France do not support an association between NBR and a higher risk of childhood acute leukemia (including ALL and AML) (102, 103).

Another talk of the special session on NBR discussed the results of a registry-based case-control study undertaken in Great Britain. This study found an excess relative risk for CL of 1.12 per mSv of cumulative red-bone-marrow dose from gamma radiation, but results for CL and radon and other childhood cancers were not significant (104). The main shortcoming of this study was the lack of individual dose assessment. As a consequence, about half of the cases and controls had the same dose-rate estimates because these were based on the mean gamma ray doses of their birth registration district. To overcome these limitations, a new study is ongoing which includes a larger number of cases and controls using an extended calendar period and an extended set of indoor gamma-ray measurements from the United Kingdom Childhood Cancer Study [UKCCS (105)]. For a better estimation of indoor gamma ray dose rates, several *ad hoc* models were explored and the results were published (106–108).

Some of the most recent studies have been summarized by Mazzei-Abba et al. (99) in 2019 and their review includes a comprehensive discussion about methodological differences, limitations, and challenges that have to be faced when evaluating the findings of these studies. They point out that larger study populations or pooled studies are needed to investigate cytogenetic subgroups of diseases, while the main challenge is to accurately assess children's individual exposure to NBR. In the latest review, Kendall et al. (109) conclude that at present no firm conclusions about NBR and childhood cancer can be reliably drawn.

### Extremely Low-Frequency Magnetic Fields (ELF-MF)

#### Epidemiological Findings

Until today, more than 40 epidemiological studies examined the relationship between ELF-MF and the risk of CL, including five pooled studies since the year 2000 (4, 110–113). A brief summary and discussion of these epidemiological findings was given in a talk. The presented study results are relatively consistent in that they show a higher risk for developing CL and specifically ALL with MF exposures above 0.3 or 0.4 μT. Based on these findings, IARC classified ELF-MF as possibly carcinogenic to humans (Group 2B) in the year 2002 (114). However, how ELF-MF may cause leukemia is unknown – until today, no plausible biological mechanism has been found, and experimental *in vitro* and *in vivo* studies do not confirm the results of the epidemiological studies. Besides, it was shown that newer epidemiological findings point to a decline in the reported relative risk (RR) since the 1990's to now (115): In their analysis, Swanson et al. (115) calculated the cumulative RR from 32 studies published up to each successive calendar year, showing that the RR settled around 2 in the mid-1990s before declining to a current value of 1.44 in 2017, though the decline is not statistically significant. Improvement of study quality was not considered an explanation for the decline by the authors. Other possible explanations for the observed decline could be a true causal risk that declined over time, as discussed by the authors, or a confounder that was present in earlier years but is not present any longer; however, such explanations would need further investigation. In a pooled analysis of the four most recently published studies, no association was observed between ELF-MF and CL (116). Combining those with results from two previous pooled analyses from Kheifets et al. (111) and Ahlbom et al. (4), attenuates the association at >0.4 μT to an odds ratio of 1.45 (95% CI: 0.95–2.20) (116). It should be pointed out that epidemiological studies on the association of ELF-MF and CL usually report on total leukemia cases and/or the ALL subtype but do not distinguish between the cytogenetic subgroups of the disease. This is due to the very small numbers of exposed cases even in the pooled analyses, but cytogenetic subgroups might display different susceptibilities to ELF-MF. Another problem faced by most epidemiological studies is the extremely low number of cases in the highest exposure category because only few children are exposed to MF ≥0.2 μT (117). In case-control studies, however, the key concern is selection bias. It was shown that non-participating controls tend to have a lower socio-economic status, which is related to higher ELF-MF exposure (118). This might have led to an overestimation of the association of the potential effect of ELF-MF on CL incidence in studies requiring subject participation (119).

#### Experimental Findings

Previous hazard identification and risk assessments by IARC, the World Health Organization (WHO), and the Scientific Committee on Emerging and Newly Identified Health Risks (SCENIHR) considered evidence from experimental animal studies on the association of ELF-MF and leukemia as inadequate (114, 120, 121), and such studies have been hampered by the fact that no animal model existed that resembled the human disease appropriately. Therefore, different mouse models and their applicability in CL research were discussed in a special session of the workshop. Presented mouse models included the *Cdkn2a* deficient/*ETV6-RUNX1* mouse model, the *PAX5-ELN* mouse model, and the Sca1-*ETV6-RUNX1* mouse model already described in section Experimental Evidence for an Infection-Mediated Childhood Leukemogenesis, *Cdkn2a* deficient/*ETV6-RUNX1* as well as *PAX5-ELN* transgenic mice developed neoplasms with a high incidence of up to 50-80%, respectively (122, 123). In case of the *Cdkn2a* deficient/*ETV6-RUNX1* mouse model also neoplasms other than B-cell lymphomas/leukemias were developed. The Sca1-*ETV6-RUNX1* mouse model was developed and tested specifically for usefulness in ELF-MF research during the EU-funded project Advanced Research on Interaction Mechanisms of electroMagnetic exposures with Organisms for Risk Assessment (ARIMMORA). As it considers the two-hit model of leukemogenesis and also shows a low penetrance, this model is most applicable to test the contribution of potential environmental risk factors as second hits. One ARIMMORA pilot project used this mouse model for studying the effect of 1.5 mT ELF-MF exposure on hematopoietic compartments of the peripheral blood in a SPF environment. A small but significant decrease in CD8^+^ T lymphocytes was seen in exposed mice at 2 months of age (124). Similarly, in another ARIMMORA project, CD1 mice exposed to 10 μT, 1 mT and 10 mT ELF-MF, respectively, showed a decrease in CD8^+^ T lymphocytes at 4 weeks of age in all exposed groups (125). If this early decrease is of any functional relevance has yet to be ascertained. Of special interest is the observation of pB-ALL development in one out of 30 Sca1-*ETV6-RUNX1* mice during the course of the above mentioned ARIMMORA pilot project (124). This finding was not statistically significant because of the small numbers of animals and it is unclear if it is connected to the decrease in CD8^+^ T lymphocytes — cells that are known mediators of anti-tumor immunity and can recognize and eliminate tumor cells (126). Future experiments with much larger animal numbers are clearly needed to validate these findings and to clarify the role of ELF-MF in the development of ALL.

### Other Environmental Factors

There are quite a number of environmental factors for leukemia that have been investigated in epidemiological studies besides IR and ELF-MF and that were not specifically addressed at the workshop [for a more detailed overview see review by Schüz and Erdmann (127)]. Among the recognized risk factors are high and low birth weight (128) and sex, with boys more often affected than girls (129). Pesticide exposure (130, 131), air pollution (132–134), paint (135), paternal tobacco smoking (136, 137), and prelabour cesarean delivery (138) have been associated with an increased risk for ALL or AML but to date none of them is regarded as an established risk factor. There are also some factors that have been associated with a reduced risk for leukemia in the child, including maternal supplementation with folic acid or vitamins (139), or breastfeeding (140). Two consortia, the Childhood Leukemia International Consortium (CLIC) and the International Childhood Cancer Cohort Consortium (I4C), are putting much effort into clarifying the relevance of the environmental risk factors in question. Nevertheless, the child's risk to develop CL can be modified by genetic susceptibility, and novel predisposition syndromes are being reported constantly. One hypothesis states that the first oncogenic hit primes the cell through epigenetic modification and such preleukemic cells could be more sensitive to environmental risk factors (49). Therefore, a multidisciplinary approach is needed in the future that considers this likely association between genetic and environmental risk factors.

## Discussion

**Figure 1:**
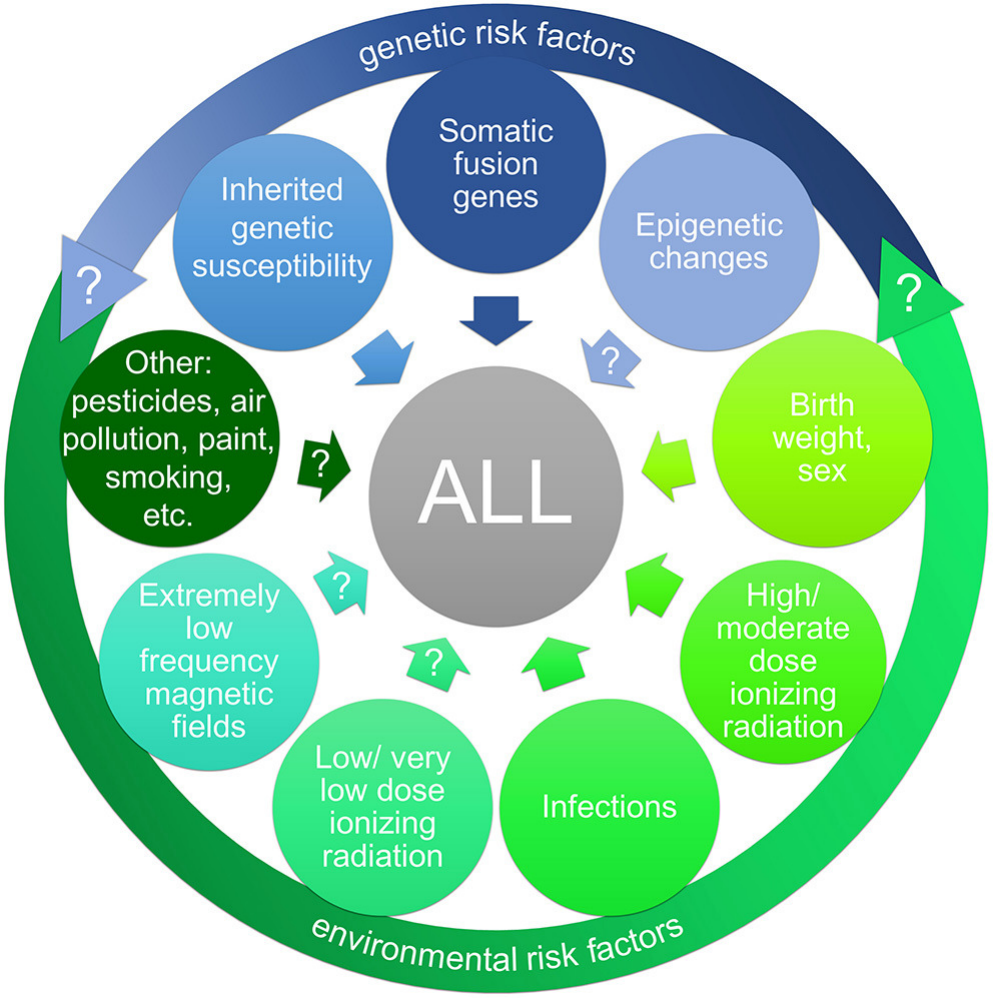
Risk factors (potential and established) for childhood acute lymphoblastic leukemia (ALL). A large body of research has targeted a wide range of possible risk factors for childhood ALL, including genetic risk factors (indicated in blue) and environmental risk factors (indicated in green). The interaction of genetic and environmental risk factors (gene-environment-interaction) may have an impact on ALL development. Inherited genetic susceptibility is detected with increasing frequency in childhood leukemia as well as preleukemic fusion genes as predisposing factors. The impact of epigenetic alterations (possibly induced by oncogenes or environmental exposures) on ALL development is yet unclear. Among the recognized environmental risk factors are high and low birth weight and sex as well as high to moderate doses of ionizing radiation. The relevance of infections as risk factor for ALL has strengthened considerably in the last decade. In contrast, evidence for an association between a higher risk for ALL and exposure to low/very low doses of ionizing radiation, extremely low frequency magnetic fields (e.g., from power lines), and other environmental risk factors (like pesticides or air pollution), respectively, has yet to be verified.

Research efforts on the causes of CL have made some progress in the past years (Figure 1). Due to new screening methods, like GIPFEL and trio sequencing, genetic predisposition is detected with increasing frequency in CL. Whereas inherited or *de novo* germline CPS are rare but highly penetrant, the presence of a somatically acquired fusion gene, like *ETV6-RUNX1*, or of a germline susceptibility locus confers only a very low or reduced disease penetrance. Regarding environmental risk factors, birth weight, sex and high to moderate doses of IR are among the recognized ones. Despite a growing body of research, results for an association between other environmental risk factors (including environmental pollutants and parental life style factors) and a higher risk for CL are largely inconsistent and overall inconclusive. One noticeable exception are infections, as evidence of a causal relationship with CL has strengthened considerably in the last years. Not only epidemiological analyses underline an association between maternal infections during pregnancy and a higher risk for CL in the offspring (77, 78). Especially two predisposed mouse models, *Pax*5^+/−^ and Sca1-*ETV6-RUNX1*, highlighted the role of infections in the development of pB-ALL (54, 79, 82). Mice of both genotypes only develop leukemia in conjunction with exposure to common pathogens and closely resemble human pB-ALL in penetrance, pathology, and genomic lesions (29, 79). These models further underscore the role of gene-environment-interactions in this disease. Such interactions may also occur through epigenetic changes induced by the environmental exposure and leading to the reprogramming of the cancer cell of origin (49). These mouse models can now be used to investigate whether and how environmental exposures other than infections affect leukemia development *in vivo*, as biological evidence is sparse. Especially in the case of ELF-MF, experimental studies are clearly needed to validate if the consistent epidemiological observation of an increased leukemia risk is real, and to identify a possible biological mechanism that could explain these findings. One promising approach may be to look for epigenetic changes after exposure to ELF-MF, as this is regarded as a potential mediating mechanism between environmental risk factors and CL.

Many other questions still remain unanswered, starting with the basic one of whether and how much geographical variation in the true incidence rates of CL there really is. Notably, within high-income countries, the incidence rates of CL vary substantially less than lifestyle- and environmentally-related cancers in adults. A multidisciplinary and global approach will be needed to bring together the existing evidence from epidemiological, experimental and mechanistic studies, to improve exposure assessment and consistency between countries, and for a better understanding of the incidence and distribution of CL worldwide. The Global Acute Leukemia network (GALnet^1^), initiated in 2010 by the BfS and coordinated by IARC, has already established an international network for a multidisciplinary study of CL, and together with the work of CLIC and I4C these international associations will hopefully provide new insights into this most common type of childhood cancer worldwide.

## Author Contributions

J-AS, GZ, SH, and JS were part of the organizing committee of the workshop. J-AS wrote the first draft of the manuscript. SH, FE, IS-G, UF, JS, and GZ contributed to the revision of the initial draft of the manuscript. All authors read and approved the final manuscript.

## Funding

The Federal Ministry for the Environment, Nature Conservation and Nuclear Safety Germany funded the international workshop (3619I02454). Open access publication fees were funded by the German Federal Office for Radiation Protection.

## Conflict of Interest

The authors declare that the research was conducted in the absence of any commercial or financial relationships that could be construed as a potential conflict of interest.

## Publisher's Note

All claims expressed in this article are solely those of the authors and do not necessarily represent those of their affiliated organizations, or those of the publisher, the editors and the reviewers. Any product that may be evaluated in this article, or claim that may be made by its manufacturer, is not guaranteed or endorsed by the publisher.

## Footnotes

^1^ https://galnet.iarc.fr
